# Understanding and therapeutically exploiting cGAS/STING signaling in glioblastoma

**DOI:** 10.1172/JCI163452

**Published:** 2024-01-16

**Authors:** Justin T. Low, Michael C. Brown, Zachary J. Reitman, Joshua D. Bernstock, James M. Markert, Gregory K. Friedman, Matthew S. Waitkus, Michelle L. Bowie, David M. Ashley

**Affiliations:** 1 Department of Neurosurgery,; 2 Department of Radiation Oncology, Duke University, Durham, North Carolina, USA.; 3 Department of Neurosurgery, Brigham and Women’s Hospital, Harvard Medical School, Boston, Massachusetts, USA.; 4 David H. Koch Institute for Integrative Cancer Research, Massachusetts Institute of Technology, Cambridge, Massachusetts, USA.; 5 Department of Neurosurgery, University of Alabama at Birmingham, Birmingham, Alabama, USA.; 6 Department of Pediatrics, Division of Pediatric Hematology and Oncology, University of Alabama at Birmingham, Birmingham, Alabama, USA.; 7 Division of Pediatrics, The University of Texas MD Anderson Cancer Center, Houston, Texas, USA.

## Abstract

Since the discovery that cGAS/STING recognizes endogenous DNA released from dying cancer cells and induces type I interferon and antitumor T cell responses, efforts to understand and therapeutically target the STING pathway in cancer have ensued. Relative to other cancer types, the glioma immune microenvironment harbors few infiltrating T cells, but abundant tumor-associated myeloid cells, possibly explaining disappointing responses to immune checkpoint blockade therapies in cohorts of patients with glioblastoma. Notably, unlike most extracranial tumors, STING expression is absent in the malignant compartment of gliomas, likely due to methylation of the *STING* promoter. Nonetheless, several preclinical studies suggest that inducing cGAS/STING signaling in the glioma immune microenvironment could be therapeutically beneficial, and cGAS/STING signaling has been shown to mediate inflammatory and antitumor effects of other modalities either in use or being developed for glioblastoma therapy, including radiation, tumor-treating fields, and oncolytic virotherapy. In this Review, we discuss cGAS/STING signaling in gliomas, its implications for glioma immunobiology, compartment-specific roles for STING signaling in influencing immune surveillance, and efforts to target STING signaling — either directly or indirectly — for antiglioma therapy.

## Introduction

**Figure 1:**
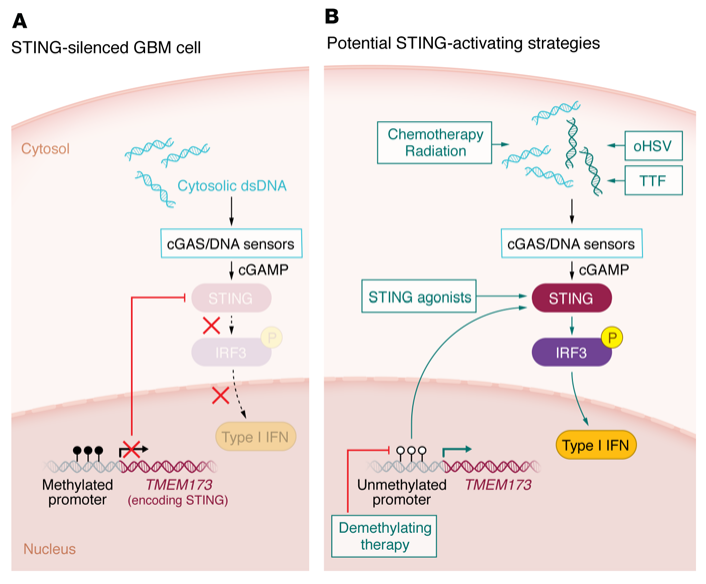
Model for STING epigenetic silencing and potential activation strategies in GBM. (**A**) The cGAS/STING pathway is silenced by *STING* promoter methylation in GBM neoplastic cells. Dashed arrows highlight loss of downstream STING signaling due to STING silencing in these cells. (**B**) Potential strategies for activating the cGAS/STING pathway are shown in green. oHSV, oncolytic herpesvirus; TTF, tumor-treating fields.

Cues from the innate immune system determine the priming, recruitment, and functionality of adaptive immunity. This crosstalk is generally mediated through antigen-independent pattern recognition receptor (PRR) signaling and results in costimulation, antigen presentation activity, and cytokine/chemokine signaling (1). Spontaneous populations of antitumor T cells have long been noted in the context of both murine and human cancers (2–5), despite the absence of apparent pathogen infection. Over the last decade, the stimulator of interferon (IFN) genes (STING) protein has emerged as a critical player in mediating endogenous PRR-induced inflammation that enables the priming of spontaneous antitumor T cell responses (6). STING senses cyclic dinucleotides (CDNs; e.g., 2′,3′-cGAMP) generated by cyclic GMP–AMP synthase (cGAS) and other viral dsDNA sensors, as well as those produced by intracellular bacteria, to induce TBK1/IRF3– and NF-κB–driven proinflammatory responses (7) (Figure 1A). DNA or CDNs from dying cancer cells can trigger STING signaling to induce antigen presentation activity, type I IFN signaling required for appropriate T cell priming by antigen-presenting cells (APCs), costimulatory ligand expression on APCs, and chemokines (e.g., CXCL9 and CXCL10) that enable T cell trafficking to the tumor site (8).

Accordingly, STING agonists were proposed to mediate antitumor immunotherapy and are being developed for clinical use — primarily as intratumoral in situ vaccines — despite initial setbacks from early clinical trials. Moreover, numerous preclinical studies have demonstrated the role of STING signaling, particularly in APCs, on tumor antigen cross-presentation and antitumor T cell immunity after various treatments, e.g., chemo/radiation (9), CD47 blockade (10), telomerase-targeting agents (11), and DNA damage (12). However, the understanding of how STING signaling occurs within the native tumor microenvironment (TME) as well as the routes by which STING signaling can be leveraged for cancer immunotherapy lack mechanistic understanding. Moreover, the expression of cGAS and STING is notably low in tumor cells from several cancer types, particularly in central nervous system (CNS) tumors (13, 14).

Here we discuss compartment-specific characteristics of STING expression and signaling in the TME; recent evidence for STING pathway suppression in the CNS; efforts to target STING therapeutically in cancer clinical trials; and potential future implications of STING modulation for the efficacy of virotherapy, standard-of-care chemo/radiation, and other modalities being investigated for targeting CNS tumors (Figure 1B).

## Compartment-specific roles for STING signaling in the TME

**Figure 2:**
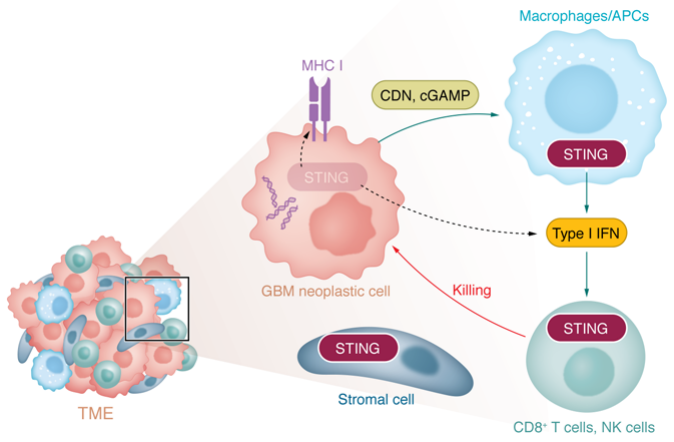
Model for compartment-specific roles for STING signaling in the GBM TME. In multiple types of cancer, the importance of STING activation has been established in both neoplastic cells and macrophages/APCs. STING activation stimulates production of type I IFNs and proinflammatory cytokines, leading to T cell– and NK cell–mediated killing of neoplastic cells. In contrast, in the GBM TME, STING is expressed in immune cells and stromal cells but not neoplastic cells. Dashed arrows indicate absence of STING pathway–mediated outcome in GBM relative to other cancers. APC, antigen-presenting cell; MHC, major histocompatibility complex; CDN, cyclic dinucleotide.

While the importance of STING signaling in priming antitumor immunity is well established, less is known about the precise contributions of STING activation in the cells comprising the tumor and TME (i.e., neoplastic cells, immune cells, and stroma/vasculature) to antitumor immunity. When implanted with intracranial GL261 murine glioma cells, mice lacking functional STING had shorter survival than wild-type counterparts, showed increased immature myeloid suppressor cells and regulatory T cells, and decreased IFN-γ^+^CD8^+^ T cells (15). In melanoma, STING signaling is necessary in APCs for spontaneous T cell priming, wherein tumor-derived DNA stimulates host APC production of type I IFNs (8) (Figure 2). In addition, selectively inducing STING signaling in dendritic cells (DCs) was also shown to engage antitumor T cells more effectively than nontargeted STING activation in mice (16). Collectively, these and other reports establish an important role for STING signaling in the TME.

Recent work also indicates roles for cGAS/STING signaling in the neoplastic compartment in dictating both cancer cell fitness and immune surveillance. KRAS/LKB1–mutant lung cancer cells suppress STING expression, wherein exogenous expression of STING led to the detection of cytoplasmic mitochondrial DNA, induction of TBK1/IRF3 innate signaling, and decreased cell fitness (17). Collectively, these findings imply that loss of STING function may suppress immunogenic and cytotoxic effects of DNA damage in cancer. In melanoma, neoplastic cell STING activation enhanced antigenicity, induced MHC class I, and improved CD8^+^ T lymphocyte–mediated killing of melanoma cells in vitro (18, 19). Similarly, STING expression in small cell lung cancer correlated with MHC class I expression and responsiveness to immune checkpoint blockade (20). Inhibition of topoisomerase resulted in tumor cell DNA damage and STING activation, which induced type I IFN responses that potentiated PD-1 blockade therapy (21). These results collectively suggest a role for neoplastic cell–intrinsic STING signaling in controlling the immunogenicity of tumor cells during the effector phase. In addition, cGAS activity in neoplastic cells may accentuate cancer cell sensitivity to DNA damage during radiation/chemotherapy (22). Release of CDNs from malignant cells can trigger STING signaling in APCs in a paracrine manner via the CDN transporter SLC19A1 (23) or by a recently described plasma membrane–localized STING isoform (24). STING activation in APCs potentially further accentuates their activation and capacity to prime T cells (Figure 2).

## STING pathway suppression in glioblastoma

STING signaling is suppressed in several cancers, including melanoma (25), colon cancer (26), and KRAS/LKB1–mutant lung cancer, perhaps as a consequence of STING’s role in antitumor immunity and sensing of DNA damage (17). STING suppression occurs through various mechanisms, including loss-of-function mutations in the genes encoding cGAS and STING (*STING1*) and by DNA methylation of their promoters (14, 17). We and others have found that in human glioma samples, *STING1* is expressed in stromal and immune cells but is uniformly suppressed in neoplastic cells (13, 27). In contrast, human vascular cells and glioma-associated myeloid cells respond to STING agonism, and STING activation in intracranial glioblastoma (GBM) murine models drives infiltration of innate immune cells, including macrophages, neutrophils, and NK cells (27). The relative importance of STING activation in specific types of T cells, NK cells, and myeloid cells remains unknown and may have important implications for designing optimal therapeutic approaches. Whether STING suppression in glioma cells contributes to the characteristically immunosuppressed nature of gliomas remains unclear.

Mutations in the *STING1* gene are rare in GBM, but methylation of a region of the *STING* promoter near the transcriptional start site is nearly universal. Interestingly, normal fetal and adult brains also exhibit *STING* promoter methylation, suggesting that epigenetic STING silencing may be characteristic of the GBM cell of origin (13). Indeed, treatment with the demethylation agent decitabine reversed *STING1* promoter methylation and rescued STING expression in GBM cell lines (13). Whether such silencing can be reversed in vivo to potentiate antitumor immunity and/or sensitize GBMs to immunotherapy, DNA damaging agents, and therapeutically delivered CDNs also remains to be determined.

## STING as a therapeutic target in GBM

Immunotherapy has yielded significant treatment success in several solid malignancies (28). However, despite favorable results in preclinical models, immunotherapeutic approaches have generally failed to improve survival over standard treatments in GBM (29, 30). This failure may be due in part to the immunologically “cold” nature of GBM, whereby spontaneous antitumor T cell responses are either absent or suppressed (31–33). The reasons for such T cell silencing are incompletely understood and are likely multifactorial; GBM tumors recruit immunosuppressive regulatory T cells (34) and myeloid cell populations (35), secrete immunosuppressive cytokines, induce T cell apoptosis, sequester T cells in the bone marrow (36), and harbor low levels of tumor-infiltrating T cells in the TME (37).

STING pathway activation may represent another approach for activating antitumor immunity given its role as a key upstream mediator of type I IFN signaling. GBM harbors extensive cytoplasmic extrachromosomal DNA (38) that could, in principle, induce the cGAS/STING pathway. However, as discussed above, STING signaling appears to be innately silenced epigenetically in the brain (13). Both direct and indirect routes to engage STING are currently being explored for glioma therapy, including STING agonists, alternating electric field therapy (e.g., tumor-treating fields [TTFs]), radiation therapy, and oncolytic viruses.

### STING agonism.

STING agonism involves the exogenous introduction of synthetic agonists designed to enhance STING signaling and the resulting IFN response. A wide range of STING agonists of varying potency and specificity have been investigated preclinically and in early-stage clinical trials for several tumor types (39, 40). Preclinical studies have demonstrated the ability of synthetic CDNs to induce tumor-specific CD8^+^ T cells (41) and reduce tumor growth when administered intratumorally in murine models in combination with immune checkpoint blockade (42). However, despite promising results in animal models, the first STING agonist to enter clinical trials, DMXAA (vadimezan), demonstrated poor efficacy against solid tumors either alone or in combination with chemotherapy (43). This disappointing outcome is potentially explained by the poor binding affinity of DMXAA for human STING despite strong binding to murine STING (44, 45). Treatment of rat esophageal adenocarcinoma models with the CDN ADU-S100 (MIW815, Novartis) resulted in stimulation of CD8^+^ T cell–mediated antitumor responses (46) and phase I clinical trial results of this agent in solid tumors demonstrated systemic immune activation (47). However, interim results from ongoing clinical trials of ADU-S100 and MK-1454 (Merck) administered intratumorally demonstrated very poor overall responses in advanced solid tumors and lymphomas (47, 48). Even when combined with pembrolizumab, MK-1454 yielded an overall response rate of only 24% (ClinicalTrials.gov NCT03172936). Multiple other intratumorally administered CDNs are currently in clinical trials. First-generation CDNs are inherently structurally unstable and generally administered intratumorally. Non-CDN STING agonists have been designed with better stability and affinity for STING to allow for systemic delivery (reviewed in ref. 39), including amidobenzimidazole-based compounds (49). Additionally, alternative approaches are in preclinical development, including bacterial vectors, antibody-drug conjugates (50), and nanoparticle vaccines (51).

The reasons for the poor clinical efficacy of STING agonists observed thus far in human trials are still being elucidated. While STING activation has primarily been studied in APCs where it stimulates IFN signaling and primes T cell responses, STING signaling can also be activated in T cells themselves, where they may activate cell stress and apoptotic pathways in addition to IFN stimulation (52). STING agonism may additionally stimulate the production of regulatory cytokines (53) and immune checkpoints (54) that actively limit antitumor responses. For example, systemic STING agonist treatment stimulates immunosuppressive B cells that suppress NK cell–mediated antitumor responses (55).

In GBM, tumor cells highly express CD47, an antiphagocytosis signal (56). Combination treatment of an anti-CD47 antibody and temozolomide induced ER stress, activated the STING pathway, and increased glioma cell phagocytosis by APCs, resulting in increased antigen cross-presentation and T cell priming (10). These results were not seen when anti-CD47 antibody was used alone. A second study demonstrated that nanoparticles encapsulating a STING agonist and coated with dual anti-CD47/anti–PD-L1 antibodies mediated robust antitumor efficacy in murine gliomas (56). These nanoparticles induced glioma-associated myeloid cell phagocytosis of tumor cells via CD47–PD-L1 ligation, and activation of T cell–supportive myeloid cell phenotypes due to STING agonist–mediated effects. Collectively, these studies suggest that STING activation in different cell populations may result in varying immunomodulatory phenotypes, and combination approaches that target specific STING regulatory programs might be required for optimal antitumor activity.

STING agonists for patients with infiltrating gliomas have not yet entered human clinical trials, although there have been initial promising results in animal models. Injection of the STING agonist c-di-GMP into the tumors of glioma-bearing mice significantly improved survival, enhanced type I IFN signaling, and increased T cell migration into the brain (15). These effects were not observed in mice homozygous for the nonfunctional Goldenticket (Gt) STING variant, establishing the necessity of STING expression in the TME. Additionally, the combination of c-di-GMP and a peripheral vaccine significantly increased survival in glioma-bearing mice, as compared with monotherapy with either c-di-GMP or peripheral vaccine alone.

Despite these promising results, the lack of spontaneous murine gliomas limits the translatability of murine results to human gliomas. This limitation is particularly important when studying the tumor immune microenvironment, which is markedly more proinflammatory in immunocompetent murine models and greatly abrogated in human-derived xenografts as compared with human gliomas. Some of these challenges may be resolved by studying spontaneous canine gliomas, whose molecular landscapes more closely resemble human gliomas (57). In a recent phase I trial, Boudreau et al. treated 5 dogs with spontaneously arising GBM with the small-molecule STING agonist IACS-8779 via intratumoral injection (58). In 3 of the 5 treated dogs, the treatment was well tolerated and reduction in the contrast-enhancing tumor volume was noted on follow-up magnetic resonance imaging (MRI). One dog, which had received the lowest dose of IACS-8779, showed tumor growth on serial MRIs following intratumor treatment. The final dog developed a fatal acute intracranial inflammatory response following intratumoral injection of IACS-8779, with postmortem evaluation showing perivascular and leptomeningeal inflammation and a mixed inflammatory polymorphonuclear leukocyte infiltrate. While small, this study provides promising support for intratumoral STING agonist treatment as a therapeutic approach for gliomas. To further advance these proof-of-concept results into the clinic, it will be important for future studies to investigate the duration of the clinical effect, optimal dose, scheduling, and potential inflammatory sequelae of STING agonism. Additionally, intratumoral administration poses technical limitations due to the need for surgery and limits the frequency of administration, while systemic administration must overcome the challenges of the blood-brain barrier and systemic inflammatory responses. Finally, the relative contribution of specific cell types of the glioma TME in mediating the clinical benefit of STING agonism is unknown and will need to be determined in future preclinical studies and larger scale trials.

### TTFs.

Alternating electric field therapy (e.g., TTFs) combined with standard-of-care temozolomide is recommended as an option by the National Comprehensive Cancer Network for the treatment of newly diagnosed GBM and as monotherapy for recurrent GBM. For newly diagnosed GBM, the EF-14 clinical trial demonstrated a median survival of 20.9 months when TTFs were used together with temozolomide, as compared with 16 months with temozolomide alone (59). For recurrent GBM, TTF monotherapy resulted in similar survival as compared to physician’s choice of chemotherapy (60). The non-uniform alternating electric fields of TTF therapy are thought to alter the spatial orientation of polar amino acids and disrupt their proper alignment at the mitotic spindle (61). This disruption ultimately inhibits tumor cell division and forms the mechanistic basis by which TTFs target dividing cancer cells (62, 63).

Recent preclinical studies have shown that TTFs can disrupt cellular membranes (64) as well as promote autophagy and ER stress (65) in addition to their known effect of disrupting mitosis. Additionally, there have been several reports of increased contrast enhancement on MRI imaging after initiation of TTF therapy followed by durable clinical and radiographic responses (66, 67). This pseudoprogression observed in some patients receiving TTF treatment has led to the hypothesis that TTFs may also induce an inflammatory response. Indeed, in patient-derived glioma stem cell lines and established human glioma lines, TTF treatment induced formation of cytosolic micronuclei clusters and activation of type I IFNs in an AIM2- and STING-dependent manner (68). In syngeneic KR158 and GL261 murine gliomas models, TTF treatment stimulated antitumor immune memory that resulted in a cure rate of 42%–66%. Paired transcriptomic analysis of PBMCs from patients with GBM before and after TTF treatment showed activation of adaptive immune signatures. These studies have motivated several clinical trials investigating the combination of TTFs with immune checkpoint blockade (69).

### Radiation therapy.

Radiation therapy (RT), long integral to the standard of care for GBM, likely exerts profound effects on the immune microenvironment. RT was established as an effective therapy for GBM with the report in 1978 that whole-brain radiation more than doubled the median overall survival (35 versus 14 weeks) (70). Subsequent studies clarified the effective dose and treatment fields (71, 72). Modern guidelines call for 60 Gy delivered in 30 daily fractions to the postsurgical resection cavity, suspected residual tumor, and areas of MRI T2–hyperintense nonenhancing tumor with a 2 to 3 cm anatomic expansion. The primary mediator of RT efficacy is thought to be production of double-strand DNA breaks, which can lead tumor cells to undergo apoptosis and/or mitotic catastrophe. However, an appreciation for RT’s effects on the immune microenvironment has recently emerged.

RT can affect the GBM immune microenvironment in several ways. RT triggers type I IFN expression through STING or through STING-independent mechanisms in a variety of in vivo models of extracranial tumors (for a review, see ref. 73). RT may promote DC and other APC activation to facilitate cross-presentation of tumor-derived antigens (74). Thus, RT might stimulate the innate immune response and stimulate antigen presentation. However, RT may deplete tumor-infiltrating lymphocytes given these cells’ intrinsic radiosensitivity (75), which could thwart adaptive immune responses. Intriguingly, several studies have shown that RT synergizes with immune checkpoint blockade in mouse models of GBM (76), suggesting that RT may play a unique role in stimulating the immune system. However, irradiation of the normal brain was found to blunt the effects of checkpoint blockade and stimulate more aggressive tumor growth in another study (77).

### Oncolytic viruses.

Oncolytic viruses (OVs) are an emerging class of immune-oncologic agents capable of promoting a robust antitumor immune response through selective tumor lysis and the induction of antitumor immunity (78, 79). OVs offer a targeted approach for the treatment of brain tumors, and as such, a litany have been tested, albeit with varying results (78, 79). Among the OVs trialed, engineered oncolytic herpes simplex virus type 1 (oHSV) has been extensively researched and several constructs have shown substantial promise in preclinical models/clinical trials in both pediatric and adult brain tumors (80–83). Of the oHSVs examined, G207 has been the most widely studied and has proven safe in the CNS of both children and adults (79–86). In addition, G207 treatment induced tumor-infiltrating lymphocytes and some prolonged responses in children with progressive high-grade glioma (83). These promising safety/efficacy data have led to a first-in-human phase I trial of intratumoral G207 in recurrent cerebellar brain tumors and the development of a multi-institutional phase II trial (ClinicalTrials.gov NCT04482933) in pediatric high-grade glioma at first relapse/progression slated to open in 2023 (87).

STING is a critical determinant of both oHSV-mediated oncolysis and the development of innate/adaptive inflammation (88). Given that PRR pathways are central to host responses for most pathogens, this role is perhaps unsurprising (89). The cGAS/STING signaling pathway detects cytosolic DNA and triggers many downstream immune responses, and in response to HSV infection induces type I IFN gene expression (90). Not surprisingly, the STING pathway is the target of a wide range of strategies utilized by herpes viruses to evade the immune response (88, 91). Thus, the STING pathway has emerged as a therapeutically relevant pathway, with a strong rationale for STING modulation in the context of oHSV-mediated therapy.

Recent evidence indicates that STING signaling is required for durable antitumor effects related to oHSV (90). However, as discussed above, primary brain tumors lack STING expression and exhibit hypermethylation of a region of the *STING* promoter (13). Interestingly, STING expression and signaling can be reconstituted in glioma cell lines via exposure to decitabine, a DNA hypomethylating agent that has been shown to enhance immune recognition and killing of glioma-initiating cells (92). Given the established role of STING signaling in cancer immunity and the potential for its modulation/reconstitution in neoplastic cells, the rational modulation of this axis may lead to therapeutic benefits when combined with oHSVs and/or RT.

However, while early and robust STING activity may antagonize oHSV infection by suppressing viral replication via IFN, STING reactivation downstream may enhance oHSV efficacy by facilitating nuclear import of HSV DNA during infection, augmenting/sustaining an antitumor immune response initiated by the virus (90, 93). Future work will therefore be required to determine the optimal timing of any combinatorial treatment strategies related to STING and oHSVs in relevant preclinical models as a translational bridge to the clinic.

## Future perspectives in STING-directed therapies

Despite the disappointing results from initial STING agonist trials, STING activation remains an enticing target for combinatorial therapeutic approaches due to its central role in priming innate antitumor immunity. The CNS environment presents additional challenges for STING activation due to its epigenetic silencing in the brain parenchyma and neoplastic cells. This epigenetic silencing could present a potential opportunity for the use of epigenetic modulation therapy to de-repress STING in neoplastic cells. Release of innate epigenetic silencing may permit the recognition of cytosolic DNA within neoplastic cells, presenting the tantalizing possibility of sensitizing gliomas to therapy-induced DNA damage, including RT, chemotherapy, and TTFs (Figure 1B). Additionally, STING de-repression may activate innate immunity and sensitize gliomas to immunotherapy, including checkpoint blockade and OVs. That STING is silenced in the normal brain parenchyma raises the question of whether de-repressing STING may lead to undesired neurotoxicity; reassuringly, however, the use of decitabine for hematologic malignancies has not resulted in significant neurotoxicity.

STING expression is regulated by negative feedback to prevent its constitutive activation. Chronic activation in fact appears to be tumorigenic in certain contexts (94–96). Thus, the degree and timing of STING activation to inflame the TME will need to be determined. Optimal STING pathway activation may require the concurrent inhibition of regulatory programs that may attenuate the impact of STING signaling and antitumor immunity, such as the antiphagocytosis signal CD47, regulatory B and T cells, and immune checkpoints. The unique immunosuppressive CNS environment means that the results from systemic cancer studies must be applied with caution to CNS tumors. The toxicity profile of STING-activating strategies in the CNS may also differ substantially from those observed in systemic cancers; a particular concern is the relative intolerance of the CNS to immune activation. Additionally, sex-specific differences in response to STING activation therapy requires further exploration; a recent publication noted significantly reduced cGAS/STING activation in females as compared with males in murine models of traumatic brain injury (97).

In this Review, we have focused on canonical STING/TBK1/IRF3–driven activation of type I IFNs. However, crosstalk between STING pathway components and other signaling nodes means that activation of STING pathway components is not necessarily proinflammatory or antitumor in all contexts. Despite its key antitumor innate immune role in mediating STING-dependent activation of type I IFNs, TBK1 contrastingly has distinct immune evasion roles that are independent of STING and IRF3. For example, TBK1 inhibition has been shown to improve the efficacy of immune checkpoint blockade in several tumor models (98). While STING is well known to activate NF-κB, it has recently been shown that NF-κB activation also activates STING via microtubule depolymerization, which prevents the trafficking of STING to lysosomes (99). Finally, STING activation may enhance the frequency of brain metastases from breast and lung cancer (100, 101).

The TME changes with disease progression and in response to treatment. An understanding of these changes could influence the optimal timing of STING activation and combinatorial regimens. However, our understanding of these dynamic processes is hampered by the paucity of patient tissue samples collected before and immediately after treatments, in particular RT, and the heterogeneity of samples analyzed at the time of recurrence. Unfortunately, murine models are unable to account for intra- and intertumoral heterogeneity, and they do not accurately model the extent of tumor evolution that occurs during standard-care therapy in GBM.

Thus, phase 0/surgical window-of-opportunity studies may be best suited to answer questions regarding STING and innate immune activation in human tumor samples.

In summary, while STING epigenetic silencing is characteristic of primary CNS tumors, it presents both a challenge to existing treatment approaches as well as a promising potential therapeutic target. Much work remains to determine how best to exploit this key innate immune pathway and design combination treatment regimens for optimal therapeutic effect.

## Version 1 01/16/2024

Electronic publication
